# When fish fails to protect: interrogating the South Asian cardiometabolic paradox in the era of aquaculture transition

**DOI:** 10.1017/S1368980026103140

**Published:** 2026-07-20

**Authors:** Abu Ansar Md Rizwan

**Affiliations:** Nutritional Sciences, Texas Tech University, USA

**Keywords:** Marine fish, *n*-3 fatty acids, South Asian, cardiometabolic risk, nutrition transition, aquaculture, heavy metals

## Abstract

South Asia presents a striking nutritional paradox: populations among the world’s highest fish consumers carry disproportionate cardiometabolic risk at BMI levels well below disease thresholds for European populations. Marine fish is established as cardioprotective in fatty-fish-consuming coastal populations, yet this benefit appears to break down in South Asian contexts. This commentary interrogates four interlocking mechanisms. First, aquaculture-driven species substitution has replaced *n*-3-rich species such as hilsa and coastal sardines with leaner farmed varieties offering only a fraction of the cardioprotective lipid profile. Second, deep frying in high-*n*-6 oils substantially degrades PUFA bioavailability. Third, heavy metal co-contamination in arsenic- and mercury-affected watersheds may attenuate *n*-3 benefits. Fourth, urbanisation is eroding fish intake among the highest-risk subgroups, while aggregate dietary data obscure this heterogeneity. Generic eat-more-fish guidance is insufficient; ethnicity-sensitive, sustainability-conscious guidance on species, preparation and contamination risk is needed to realise fish’s cardioprotective potential in South Asia.

Consider a country where fish is served at virtually every meal, where children grow up eating it daily and where it constitutes nearly 60 % of all animal protein consumed, yet where heart disease strikes a decade earlier than in populations that eat far less fish^(1,2)^. Bangladesh is such a country. So are Sri Lanka, coastal India and West Bengal. South Asian populations are among the world’s highest per capita consumers of fish and aquatic foods, yet they carry some of the most elevated cardiometabolic risk profiles globally relative to BMI, manifesting type 2 diabetes, dyslipidaemia and coronary artery disease at BMI levels considered normal by conventional standards^(3)^. This is not a trivial inconsistency. It is a paradox that challenges the foundational assumptions of fish-based dietary guidance and demands a food-systems-level explanation. The cardioprotective properties of marine fish, principally attributed to long-chain *n*-3 PUFA, EPA and DHA, are well documented. These fatty acids reduce plasma TAG, lower resting heart rate, attenuate vascular inflammation and improve endothelial function^(4)^. Populations in Japan, Greenland and the Mediterranean have historically demonstrated cardiovascular benefits consistent with high marine fatty acid intake. Yet these observations do not translate uncritically to South Asian contexts, where critical differences in species, preparation and contaminant burden may systematically nullify this benefit.

Fish consumption in Bangladesh, Sri Lanka and coastal and riverine India is not a dietary supplement, and it is the dietary anchor. In Bangladesh, fish covers approximately 60 % of total annual animal protein intake, with per capita daily consumption exceeding 60 g^(1)^. The hilsa (*Tenualosa ilisha*), the national fish and a migratory estuarine species indigenous to the Bay of Bengal, historically constituted the fatty fish backbone of Bangladeshi diets, providing EPA and DHA levels comparable to North Atlantic oily fish in peak season^(5–7)^. In Sri Lanka and coastal India, sardines, mackerel and anchovies fulfil an analogous nutritional role. Yet cardiometabolic epidemiology tells a contradictory story. South Asians, encompassing populations from Bangladesh, India, Pakistan, Sri Lanka and Nepal, exhibit a characteristic ‘thin-fat’ adiposity phenotype, disproportionately high visceral and ectopic adiposity relative to lean mass even at low BMI, driving insulin resistance, atherogenic dyslipidaemia and early-onset type 2 diabetes^(3)^. Cardiovascular events occur approximately a decade earlier in South Asians than in European-ancestry populations, with weaker correlations to conventional BMI-based risk thresholds^(8)^. The question this paradox demands is not whether fish is healthy, as the mechanistic evidence is robust, but why the theoretical benefit is not being realised in populations that consume it habitually.

## Mechanism 1: aquaculture-driven species substitution and *n*-3 dilution

The single most consequential but least-discussed factor is what has happened to the fish itself. Over the past three decades, rapid aquaculture expansion across South Asia has fundamentally transformed the composition of fish species available to consumers. Farmed freshwater species, tilapia (*Oreochromis niloticus*), pangasius (*Pangasianodon hypophthalmus*) and silver carp, now dominate urban and peri-urban retail markets across Bangladesh, India and Pakistan^(9)^. These species are nutritionally distant from the fatty coastal and estuarine fish they have displaced. Farmed tilapia provides approximately 100–150 mg of combined EPA and DHA per 100 g, roughly 10–15 % of what fatty species such as hilsa or Atlantic mackerel deliver^(10)^. Pangasius offers a similarly impoverished *n*-3 profile, with *n*-6׃*n*-3 ratios far exceeding cardioprotective ranges^(11)^. The epidemiological literature linking fish consumption to cardiovascular benefit draws overwhelmingly on populations consuming fatty marine species, salmon, herring, mackerel and sardines, with EPA + DHA concentrations of 1–3 g per 100 g serving^(4)^. A South Asian population that records high fish consumption frequency in dietary surveys but is predominantly eating farmed tilapia is not receiving *n*-3 exposures that replicate those modelled in that literature. Dietary assessment tools that aggregate ‘fish’ as a homogeneous category systematically overestimate cardioprotective lipid intake. This epidemiological conflation may be partly responsible for the apparent paradox: the fish being eaten is not the fish the guidelines assume.

## Mechanism 2: cooking practices as a vector of *n*-3 destruction

Even when fatty fish are consumed, dominant South Asian preparation methods substantially reduce the bioavailability of *n*-3 PUFA before they reach the body. Deep frying, standard practice across Bangladesh, Pakistan and large parts of India, has been shown to reduce EPA and DHA content by 70–85 % in studies of tuna and other fatty fish, primarily through thermal oxidation and fatty acid migration into frying oils^(12)^. Extended cooking duration, high temperatures and repeated oil use, all characteristic of home and street-food frying in South Asian contexts, amplify this effect. Oxidised *n*-3 fatty acids produced through these processes are not merely neutral; they may actively promote rather than inhibit oxidative stress and endothelial dysfunction^(13)^. A population consuming fried fish at high frequency may therefore be receiving a fatty acid profile that is pro-inflammatory at the point of consumption, notwithstanding the inherent nutritional quality of the raw ingredient. Fish consumption research rarely captures preparation methods, rendering the cardioprotective assumption of fish intake doubly misleading.

## Mechanism 3: heavy metal co-contamination as a cardiometabolic confounder

The rivers and coastal waters supplying the majority of South Asian fish consumption are among the most contaminated aquatic environments in the world. Arsenic contamination, primarily from groundwater mobilisation and agricultural runoff, affects an estimated 50 % of tube wells in Bangladesh and contaminates fish raised in irrigated aquaculture ponds^(14)^. Chronic arsenic exposure has been associated with elevated fasting glucose, insulin resistance, metabolic syndrome and hypertension in Bangladeshi cohorts, independent of conventional cardiometabolic risk factors^(15)^. Mercury contamination in riverine fish from industrial discharge zones adds a parallel burden, with methylmercury documented to disrupt mitochondrial function and independently elevate cardiovascular risk^(16)^. The critical implication for the fish paradox is one of co-exposure, the *n*-3 benefit and the heavy metal harm arrive in the same food vehicle. Studies in other high-consuming populations have demonstrated dose-dependent attenuation of the expected cardiovascular benefit from *n*-3 PUFA by concurrent methylmercury exposure^(16)^. For South Asian populations consuming locally sourced fish from arsenic-affected watersheds, particularly in the Ganges–Brahmaputra delta and coastal industrial zones, this co-exposure may substantially offset the cardioprotective potential of habitual fish consumption. This mechanism is almost entirely absent from current dietary guidance frameworks for these populations.

## Mechanism 4: urbanisation eroding fish intake in the highest-risk subgroups

The fourth mechanism reverses the paradox’s logic; rather than fish failing to protect, it may simply not be reaching the people who most need it. Urbanisation across South Asia is proceeding at an unprecedented pace, and it is consistently associated with dietary transition away from traditional fish-centred patterns towards ultra-processed, energy-dense foods^(17)^. Evidence from Bangladesh and India indicates that urban residents, precisely the demographic experiencing the steepest rise in cardiometabolic disease burden, consume fish less frequently and in smaller quantities than rural counterparts, despite higher incomes, driven by time constraints, loss of traditional market access and the proliferation of convenience food alternatives^(9)^. This creates a structural epidemiological problem: aggregate national dietary data report high fish consumption for South Asian populations, while the heaviest cardiometabolic burden falls in urbanising subpopulations where fish intake is declining most rapidly. Cross-sectional analyses using population-wide dietary averages, therefore, overestimate fish-derived *n*-3 exposure in the groups carrying the greatest disease risk. Disaggregation by urban–rural status and income quartile is essential for any meaningful fish–cardiometabolic analysis in these populations.

## Implications for dietary guidance and food policy

Current WHO dietary guidelines and national guidelines across South Asian countries advise increased fish consumption as a source of beneficial fats and protein, without specifying species, preparation method or contamination risk^(18)^. This commentary argues that such guidance is not merely insufficient but may be actively misleading in contexts where species substitution, frying practices and heavy metal exposure are the operative reality for most consumers. Three concrete adjustments are warranted. First, dietary guidance for South Asian populations should specify fatty, *n*-3-dense species, such as hilsa, sardines, mackerel and anchovies, rather than endorsing fish generically. As aquaculture has made lean farmed species the affordable default in urban markets, generic ‘eat more fish’ messaging may paradoxically encourage substitution towards nutritionally inferior options. Second, the preparation method must be addressed. Guidance promoting steaming, light grilling or shallow frying with minimal oil would preserve substantially more *n*-3 PUFA at the point of consumption than current practice. Third, for populations in arsenic- and mercury-affected watersheds, which encompass the majority of riverine Bangladesh and parts of coastal India, guidance should address species selection to minimise heavy metal co-exposure, prioritising open-water marine species over bottom-feeding freshwater species where accessible and economically feasible. Beyond dietary guidance, the paradox exposed here has implications for aquaculture governance. The rapid displacement of nutritionally superior native species by high-yield but *n*-3-poor farmed varieties is a food systems failure with quantifiable cardiometabolic consequences. These same aquaculture practices raise broader environmental sustainability concerns, including pressure on wild fish stocks, fishmeal-dependent feed systems and the ecological costs of intensive pond aquaculture, that must be weighed alongside nutritional goals when designing dietary guidance and food policy for South Asia^(19,20)^.

Policy mechanisms that incentivise the production and affordability of fatty native species, including hilsa conservation programmes and *n*-3-enriched aquaculture feed standards, would address the paradox at its structural source rather than its individual symptom.

## Research gaps and future directions

Prospective studies in South Asian populations should collect species-disaggregated fish consumption data and preparation method alongside validated biomarkers of *n*-3 status, erythrocyte EPA + DHA percentage, rather than relying on fish frequency alone. Heavy metal biomarkers, including urinary arsenic and blood methylmercury, should be incorporated into studies examining fish–cardiometabolic associations in contaminated watershed populations. Whether the *n*-3 threshold required to modify visceral adiposity and insulin resistance differs in the thin-fat South Asian phenotype relative to European-ancestry populations remains untested and represents a priority question. Intervention studies substituting farmed lean species with traditional fatty species would provide the causal evidence needed to translate this argument into actionable guidance.

## Conclusion

The South Asian fish paradox is not an anomaly; it is the predictable consequence of applying dietary guidance developed in one nutritional context to a population operating within an entirely different food system. When the fish has changed species, when cooking destroys the benefit, when contamination counteracts the protection and when the highest-risk subgroups are eating less fish than aggregate data suggest, divergence between consumption statistics and cardiometabolic outcomes is expected. Resolving this requires a fundamental shift, from fish consumption frequency as a dietary metric to fish quality, preparation and safety as the operative determinants of nutritional value. Until that shift reaches evidence, guidance and food policy, one of the world’s most cardiometabolically vulnerable populations will continue to derive less protection from one of its most important traditional foods than the literature would predict.
